# Precision Stereotactic Body Radiotherapy for Ultra‐Central NSCLC: Tailored Margins Reduce Toxicity Without Compromising Tumor Control

**DOI:** 10.1002/mco2.70589

**Published:** 2026-07-22

**Authors:** Di Liu, Shuangyan Yang, Yun Chen, Ming Liu, Ruifeng Zhao, Minren Hu, Bin Su, QiongYa Wu, Hongyu Wu, Hui Liu, Jinming Yu, Yaping Xu

**Affiliations:** ^1^ Department of Radiation Oncology Shanghai Pulmonary Hospital School of Medicine Tongji University Shanghai China; ^2^ Department of Radiation Oncology Shandong Cancer Hospital and Institute Shandong First Medical University and Shandong Academy of Medical Sciences Jinan China

**Keywords:** efficacy, non‐small cell lung cancer, safety, stereotactic radiotherapy, ultra‐central

## Abstract

Stereotactic body radiation therapy (SBRT) is effective for early‐stage non‐small cell lung cancer (NSCLC), but the treatment of ultra‐central NSCLC (UCNLC) near critical structures remains challenging. This retrospective study analyzed 66 patients with stage I–II unresectable UCNLC who underwent SBRT at Shanghai Pulmonary Hospital (2020–2023). UCNLC was defined by planning target volumes (PTVs) abutting/overlapping vital structures (proximal bronchial tree, esophagus, heart, great vessels, pulmonary vessels) or internal target volumes (ITVs) within 1 cm of these organs. Tailored SBRT plans with non‐uniform PTV margins were used in 45.5% of cases, enabling 53.3% of patients to receive a biologically effective dose ≥ 100 Gy. With a median follow‐up of 32.2 months, the median progression‐free survival (PFS) was 42.9 months; 1‐ and 3‐year PFS rates were 93.9% and 68.8%, respectively. Median local control (LC) and overall survival (OS) were not reached, with 1‐ and 3‐year LC rates of 98.5% and 84.6%, and OS rates of 98.5% and 88.7%, respectively. Toxicities were predominantly grade 1–2, with only one grade 3 pneumonitis. Larger ITV predicted poorer LC and PFS, whereas T stage independently predicted PFS and OS. Therefore, a non‐uniform PTV strategy maintained clinical efficacy without compromising safety, supporting its application in UCNLC management.

## Graphical abstract

A tailored non‐uniform PTV strategy (including PTVcrop and PTV_high/PTV_low) was used in SBRT for ultra‐central NSCLC to meet stringent OAR constraints. Notably, this SBRT strategy achieved favorable clinical outcomes, with an 84.6% 3‐year local control rate, a median PFS of 42.9 months. Additionally, treatment‐related toxicity was mild, with only 1.5% of patients experiencing grade 3 pneumonitis and no grade 5 adverse events observed.

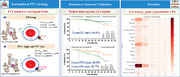

## 1 Introduction

Stereotactic body radiotherapy (SBRT) is an established standard‐of‐care for patients with inoperable early‐stage non‐small cell lung cancer (NSCLC), particularly those with peripheral tumors. Its ability to deliver high radiation doses with precision while sparing surrounding healthy tissues has translated into excellent local control (LC) and survival outcomes [1, 2, 3]. However, the management of central and ultra‐central tumors—those located in proximity to critical structures—remains challenging due to the significantly higher risk of severe toxicities. Timmerman et al. first reported that patients receiving 60–66 Gy in three fractions for centrally located tumors (within 2 cm of the proximal bronchial tree [PBT]) faced an 11‐fold increased risk of severe toxicities compared to those with peripheral tumors [4]. Consequently, the central airway has been traditionally regarded as a “no‐fly zone” for SBRT [5]. Subsequent studies, however, demonstrated that carefully adapted dose fractionation and stringent organ‐at‐risk (OAR) constraints can mitigate such risks while maintaining clinical efficacy [6]. For instance, the RTOG 0813 trial investigated dose escalation from 50 to 60 Gy in five fractions for central tumors and reported a manageable incidence of severe (grade ≥ 3) toxicities at 7.2% [7]. Similarly, our institution's prior studies showed that delivering a biologically effective dose (BED_10_) of 100–119 Gy over 4–10 fractions to centrally located NSCLC achieved a 3‐year overall survival (OS) rate of 85.3%, with severe toxicities limited to 6.5% [8]. Collectively, these findings underscore that, with meticulous planning and dose optimization, SBRT can be safely and effectively extended to treat centrally located tumors.

For ultra‐central NSCLC (UCNLC)—those abutting or overlapping vital structures such as the PBT, esophagus, or major pulmonary vessels—the risk remains significantly higher when using SBRT. Various fractionation regimens for SBRT in UCNLC have been explored, yet treatment‐related mortality rates remain substantial, ranging from 5% to 21%, particularly for tumors within 1 cm of the trachea or mainstem bronchi [9, 10, 11]. Thus, the challenge in UCNLC management lies in achieving an optimal balance between therapeutic efficacy and safety. The first prospective trial for ultra‐central tumors, the HILUS study, assessed the safety and efficacy of delivering 56 Gy in eight fractions to tumors situated within 1 cm of the PBT [12]. Unfortunately, the toxicity profile was notably concerning, with 33.8% (22/65) of patients experiencing grade 3–5 toxicities, including a 15.4% incidence of grade 5 events. Fatal bronchopulmonary hemorrhages occurred in eight patients with tumors abutting the main or lobar bronchi [12]. These findings highlight the urgent need for innovative approaches to reduce SBRT‐associated toxicities in patients with UCNLC, without compromising the substantial therapeutic benefits of this modality.

Balancing dose intensity with safety remains a fundamental challenge in SBRT for UCNLC [13]. Two primary strategies have emerged to address this challenge: increasing the number of fractions to reduce the BED_10_ and implementing more conservative constraints for OAR. The LungTech phase II trial, for instance, employed a regimen of 7.5 Gy × 8 fractions for centrally and ultra‐centrally located tumors—defined as those within 1 cm of the mainstem bronchi or 2 cm of the trachea. However, doses of up to 60 Gy were permitted even when tumors abutted or overlapped critical structures such as the bronchus, resulting in severe toxicities, including grade 5 hemoptysis [14]. In contrast, the SUNSET study applied a more cautious approach, rigorously limiting PBT invasion, minimizing planning target volume (PTV) expansion, and restricting maximum dose hotspots. This approach markedly reduced severe toxicity, with only one patient (3.3%) experiencing grade 5 toxicity (fatal interstitial pneumonia), thus renewing optimism for the safe use of SBRT in UCNLC [15].

**FIGURE 1:**
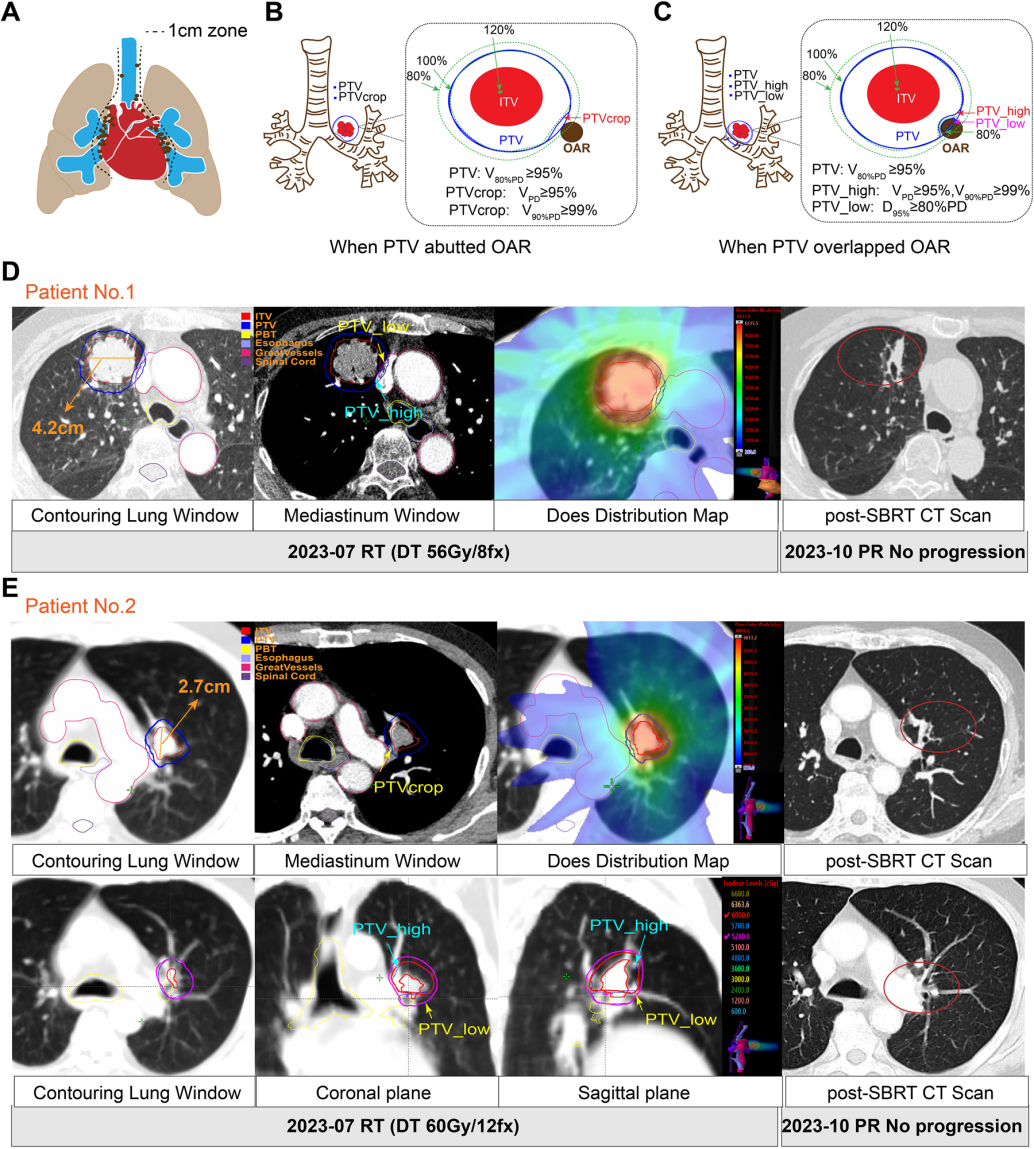
Illustration of non‐uniform PTV compromise: PTVcrop and PTV_high/PTV_low. (A) A simplified illustration of the lungs, created using Adobe Illustrator, features a black dotted line representing the 1 cm outline of central OARs. This outline is used to simplify the representation of ultra‐central tumor locations in our cohort. Brown dots indicate the distribution of ultra‐central tumors within this defined zone. The central structures in our cohort include the PBT—comprising the trachea, carina, mainstem bronchi, and lobar bronchi—as well as the esophagus, heart, great vessels (superior vena cava, aortic arch, and aorta), and pulmonary vessels (pulmonary artery and vein). (B) When PTV abutted central OARs, the PTV was modified by subtracting the OAR with an expansion margin of 1–3 mm, creating a cropped target volume referred to as PTVcrop. The prescription isodose surface was optimized to ensure that 95% of the PTVcrop was encompassed by the prescription isodose line, and 99% of the PTVcrop receives at least 90% of the prescribed dose. At least 95% of the PTV had to receive at least 80% of the prescribed dose. (C) When the PTV overlapped with central OARs, it was divided into two sub‐volumes: PTV_high and PTV_low. For PTV_high, 95% of the volume was covered by the prescription isodose line, and 99% received at least 90% of the prescribed dose. For the PTV_low, 95% of the volume received at least 80% of the prescribed dose. The maximum dose within the PTV_high was constrained to approximately 120% at the normalization point (100%). (D) Patient No. 1 was a 76‐year‐old female whose PTV overlapped with the superior vena cava. The PTV was divided into PTV_high (light blue arrow) and PTV_low (yellow arrow). The patient received SBRT to a total dose of 56 Gy in 8 fractions. From left to right, the panels show the lung‐window contouring CT image, the mediastinal‐window image, the dose distribution map (with a 3D reconstruction shown in the inset at the upper right), and CT images obtained 3 months after SBRT. The patient achieved a partial response, and no severe toxicity was observed. (E) Patient No. 2 was a 77‐year‐old male whose PTV abutted the left pulmonary artery and overlapped the proximal bronchial tree (PBT; left upper lobe bronchus). When abutting the left pulmonary artery, the PTVcrop approach was applied (upper row; yellow arrow in the mediastinal‐window image), and the patient received SBRT to a total dose of 60 Gy in 12 fractions. In the region overlapping the PBT, a PTV_high/PTV_low strategy was used (lower row); the PTV_low dose corresponded to a BED of 76 Gy. Upper row (left to right): the lung‐window contouring CT image, the mediastinal‐window image, the dose distribution map (with a 3D reconstruction shown in the inset at the upper right), and CT images obtained 3 months after SBRT. Lower row (left to right): lung‐window CT images showing contouring of the tumor region overlapping the PBT, including axial, coronal, and sagittal views, followed by CT images obtained 3 months after SBRT. The post‐treatment images demonstrate a partial response. CT, computed tomography; DT, dose to target; ITV, internal target volume; OAR, organ at risk; PD, prescribed dose; PR, partial response; PTV, planning target volume; RT, radiation therapy; SBRT, stereotactic body radiation therapy; V, volume;.

Recent systematic review and the American Radium Society (ARS) guidelines emphasize the critical need for individualized fractionation regimens, advanced intensity‐modulated radiation therapy (IMRT) techniques, and proactive OAR protection to optimize the therapeutic ratio in central and UCNLC. Nonetheless, these guidelines lack detailed and standardized dose optimization strategies, underscoring an unmet clinical need [16]. At our institution, we have uncompromisingly applied stringent OAR constraints initially designed for peripheral tumors to ultra‐central cases (Table S1). To satisfy these rigorous requirements, we implemented a non‐uniform PTV strategy using either a PTVcrop or a PTV_high/PTV_low planning approach (Figure 1). This strategy allowed us to safely administer potentially curative SBRT to patients who would otherwise have been deemed ineligible for this treatment. Here, we detail our clinical experiences and analyze treatment outcomes to assess the safety and efficacy of this tailored SBRT approach for UCNLC.

## 2 Results

### 2.1 Patients and Tumor Dosimetry Characteristics

A total of 66 patients were included in the analysis. The median age was 72 years (range: 43–93 years), with a majority of male patients (63.6%, *n* = 42). Most patients exhibited favorable performance statuses, with 93.9% presenting an ECOG score of 0 or 1. The median tumor size was 2.3 cm (range: 0.6–4.4 cm), and T1‐stage tumors represented the majority (71.2%, *n* = 47). The median PTV was 29.7 cc (95% CI: 24.1–37.0 cc), and the median ITV was 10.3 cc (95% CI: 7.5–13.5 cc). Among ultra‐central tumors, 41 (62.1%) overlapped with one critical structure, while 25 (37.9%) overlapped with two or more critical structures. The predominant dose‐fractionation scheme was 50 Gy delivered in five fractions (56.1%), followed by 60 Gy in 12 fractions (6.1%). The median BED_10_ was 100 Gy (range: 72–105 Gy), with 48 patients (72.7%) receiving ≥ 90 Gy and 39 patients (59.1%) achieving ≥ 100 Gy. The highest prescribed BED_10_ was 105 Gy. Eighteen patients (27.3%) received less than 90 Gy of radiation. Non‐uniform PTV compromises were implemented in 30 patients (45.5%) to adhere to stringent OAR constraints, enabling 53.3% of these patients to still receive the standard regimen of 50 Gy in five fractions. Notably, the lowest BED_10_ prescribed within the non‐uniform PTV subgroup was 72.8 Gy. A detailed summary of baseline patient and dosimetric characteristics is provided in Table 1.

**TABLE 1:** Patient and tumor characteristics.

Characteristics	*N* = 66 (%)
Age, years	
Median (range)	72 (43–93)
Sex, *n* (%)	
Male	42 (63.6)
Female	24 (36.4)
Smoking status, *n* (%)	
Never	46 (69.7)
Past or current	20 (30.3)
Histology, *n* (%)	
Adenocarcinoma	28 (42.4)
Squamous carcinoma	9 (13.6)
Others^a^	29 (44.0)
ECOG, *n* (%)	
0	33 (50.0)
1	29 (43.9)
2	4 (6.1)
T stage, *n* (%)	
T1	47 (71.2)
T2	19 (28.8)
T3	0
Median tumor size (cm), range	2.3 (0.6–4.4)
BED_10_ prescribed dose, Gy	
< 90Gy	18 (27.3)
≥ 90Gy	48 (72.7)
PTV location (single organ involvement), *n* (%)	41 (62.1)
Overlap with the proximal bronchial tree	18 (27.3)
Overlap with esophagus	1 (1.5)
Overlap with pulmonary vessels	11 (16.7)
Overlap with heart	5 (7.6)
Overlap with great vessels	6 (9.1)
PTV location (involving ≥ 2 organs), *n* (%)	25 (37.9)
Overlap with the proximal bronchial tree and the esophagus	2 (3.0)
Overlap with the proximal bronchial tree and pulmonary vessels	5 (7.6)
Overlap with proximal bronchial tree and Great vessels	1 (1.5)
Overlap with esophagus and great vessels	1 (1.5)
Overlap with pulmonary vessels and great vessels	8 (12.1)
Overlap with the proximal bronchial tree, pulmonary vessels, and esophagus	2 (3.0)
Overlap with proximal bronchial tree, pulmonary vessels, and great vessals	6 (9.1)
PTV compromise	
None	36 (54.5)
PTVcrop	4 (6.1)
PTV_high/PTV_low	26 (39.4)

^a^ Others refers to NSCLC, not otherwise specified (NOS).

Abbreviations: BED_10_, Biologically effective dose (with α/β ratio = 10); ECOG, Eastern Cooperative Oncology Group; PTV, planning target volume.

### 2.2 Efficacy and Toxicities

**FIGURE 2:**
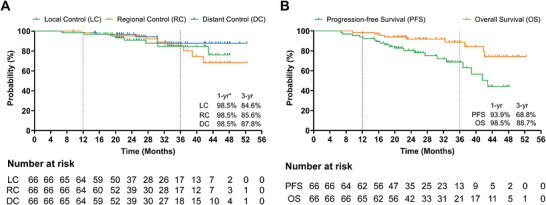
The efficacy of SBRT in managing UCNLC. Kaplan–Meier plots illustrating clinical outcomes for all patients treated with SBRT for UCNLC. (A) Local control, regional control, and distant control. (B) Progression‐free survival and overall survival. *yr = year rates. DC, distant control; LC, local control; OS, overall survival; PFS, progression‐free survival; RC, regional control.

Over a median follow‐up of 32.2 months (range, 9.6–52.4 months), the median duration of LC was not reached. The 1‐ and 3‐year LC rates were 98.5% and 84.6%, respectively (Figure 2A). During follow‐up, eight patients developed local recurrences, with the earliest recurrence documented at 6.7 months post‐SBRT (Table S2). Six patients experienced regional recurrence within 3 years, corresponding to 1‐ and 3‐year RC rates of 98.5% and 85.6%, respectively (Figure 2A). Additionally, five patients developed distant metastases, resulting in 1‐ and 3‐year DC rates of 98.5% and 87.8%, respectively (Figure 2A). The median PFS was 42.9 months (95% CI: 36.1–49.7 months), with 1‐ and 3‐year PFS rates of 93.9% and 68.8%, respectively. Median OS was not reached, and the 1‐ and 3‐year OS rates were 98.5% and 88.7%, respectively (Figure 2B, Table S3). Regarding tumor response, partial responses were observed in 46 patients (69.6%), and complete responses occurred in six patients (9.1%), resulting in an overall disease control rate (DCR) of 100% (Table S3).

Comprehensive evaluation of acute and long‐term treatment‐related toxicities involving the lung, PBT, esophagus, heart, and vascular structures was performed. Pneumonitis was identified as the most common acute toxicity, affecting 36.4% of patients (grade 1–2), with only one case (1.5%) progressing to grade 3 pneumonitis. Fatigue and anxiety (grade 1) were reported in approximately 9% of patients during the acute phase. Long‐term toxicity predominantly included pulmonary fibrosis, reported in 10.6% of patients at grade 1–2, without any instances of grade 3 or higher fibrosis. Importantly, no grade 5 treatment‐related toxicities were recorded (Table 2, Figure S1). Eight patients died from causes unrelated to treatment, including cerebral hemorrhage, chronic obstructive pulmonary disease, brain metastasis, cardiac failure, and leukemia. Collectively, these findings demonstrate that SBRT for UCNLC was well‐tolerated, displaying a favorable safety profile and promising efficacy outcomes at our institution.

**TABLE 2:** The acute and long‐term toxicities associated with SBRT in UCNLC.

Toxicity	Acute—Grade (%)					Long‐term—Grade (%)				
	G1	G2	G3	G4	G5	G1	G2	G3	G4	G5
Anxiety	6 (9.1)	2 (3.0)	—	—	—	—	—	—	—	—
Atelectasis	1 (1.5)	—	—	—	—	1 (1.5)	—	—	—	—
Cough	2 (3.0)	2 (3.0)	—	—	—	1 (1.5)	—	—	—	—
Dermatitis (radiation)	2 (3.0)	—	—	—	—	—	—	—	—	—
Dry mouth	1 (1.5)		—	—	—	—	—	—	—	—
Dyspnea	2 (3.0)	1 (1.5)	—	—	—	1 (1.5)	—	—	—	—
Esophagitis	2 (3.0)	1 (1.5)	—	—	—	—	—	—	—	—
Fatigue	6 (9.1)	3 (4.5)	—	—	—	2 (3.0)	—	—	—	—
Fever	2 (3.0)	—	—	—	—	—	—	—	—	—
Headache	1 (1.5)	—	—	—	—	—	—	—	—	—
Myalgia	1 (1.5)	—	—	—	—	—	—	—	—	—
Pain	1 (1.5)	—	—	—	—	—	—	—	—	—
Pleural effusion	1 (1.5)	—	—	—	—	1 (1.5)	—	—	—	—
Pneumonitis	19 (28.8)	5 (7.6)	1 (1.5)	—	—	2 (3.0)	—	—	—	—
Pulmonary fibrosis	—	—	—	—	—	6 (9.1)	1 (1.5)	—	—	—
Ventricular arrhythmia	1 (1.5)	—	—	—	—	1 (1.5)	—	—	—	—

Abbreviations: G1, Grade1; G2, Grade 2; G3, Grade 3; G4, Grade 4; G5, Grade 5; SBRT, stereotactic body radiation therapy; UCNLC, ultra‐central non‐small cell lung cancer.

### 2.3 Impact of Non‐Uniform PTV Strategy on Clinical Outcomes

To investigate whether a non‐uniform PTV strategy influences LC, PFS, and OS, we divided the cohort into two subgroups: a non‐uniform PTV compromise subgroup (*n* = 30; including four patients with PTVcrop and 26 with PTV_high/PTV_low strategies) and a standard (normal) PTV subgroup (*n* = 36). Dose–volume histogram (DVH) analysis revealed reduced overall target coverage in the non‐uniform PTV subgroup compared to the standard PTV subgroup (Figure S2). However, despite these differences in target coverage, there were no significant differences in LC, PFS, or OS between the two groups (Figure S3A–C). Both groups exhibited comparable 1‐ and 3‐year LC rates (non‐uniform PTV: 100% and 83% vs. standard PTV: 97.2% and 84.1%; *p* = 0.45, Figure S3). Interestingly, subgroup analysis of patients with larger tumors (T2‐stage) revealed that the non‐uniform PTV strategy significantly improved LC (*p* = 0.045). However, it did not have a significant effect on PFS or OS (Figure S3D–F).

The rationale behind adopting the non‐uniform PTV strategy was to enable higher BED_10_ delivery in patients with UCNLC. Within the non‐uniform PTV subgroup, most patients (83.3%, 25/30) received a BED_10_ of ≥ 90 Gy, with more than half (53.3%, 16/30) achieving a BED_10_ of ≥ 100 Gy. In contrast, a higher proportion of patients in the standard PTV subgroup (36.1%, 13/36) received a BED_10_< 90 Gy, which was numerically associated with shorter PFS and OS (Figure S4).

### 2.4 Identification of Prognostic Factors

We performed univariate and multivariate Cox regression analyses to identify prognostic factors for LC, PFS, and OS among UCNLC patients treated with SBRT. In the univariate analyses, ITV and PTV showed significant correlation with LC, while T stage, ITV, and PTV were predictive of PFS (Table 3). Multivariate analysis subsequently confirmed that ITV was an independent predictor of LC, whereas T stage independently predicted PFS (Table 3). Regarding OS, univariate and multivariate analyses revealed smoking status, ECOG PS, T stage, and BED_10_ (< 90 Gy or ≥ 90 Gy) as significant predictors (Table 3).

**TABLE 3:** Univariate and multivariate Cox regression analysis for LC, PFS, and OS.

Characteristics	Univariate analysis						Multivariate analysis					
	LC		PFS		OS		LC		PFS		OS	
	HR (95% CI)	*p* value	HR (95% CI)	*p* value	HR (95% CI)	*p* value	HR (95% CI)	*p* value	HR (95% CI)	*p* value	HR (95% CI)	*p* value
Age							—					
≤ 72	1		1		1							
> 72	1.79 (0.43–7.51)	0.42	1.52 (0.62–3.73)	0.36	1.07 (0.99–1.16)	0.10						
Sex	0.98 (0.48–2.01)	0.96	1.38 (0.83–2.29)	0.22	6.52 (0.38–110.95)	0.20	—					
Smoking status	0.84 (0.41–1.73)	0.64	0.66 (0.43–1.03)	0.07	0.38 (0.17–0.83)	0.016^*^	—		—		0.26 (0.09–0.76)	0.014^*^
Histology							—					
Adenocarcinoma	1	0.96	1	0.35	1	0.42						
Squamous	1.23 (0.29–5.34)	0.78	0.61 (0.31–1.21)	0.15	3.18 (0.52–19.42)	0.21						
Others^#^	—	0.99	1.38 (0.59–3.19)	0.46	1.11 (0.24–5.08)	0.89						
ECOG	0.49 (0.13–1.90)	0.31	1.51 (0.71–3.23)	0.29	3.47 (1.17–10.33)	0.025^*^	—		—		3.46 (1.19–10.04)	0.022^*^
T stage	3.39 (0.83–13.86)	0.09	4.21 (1.73–10.26)	0.002^**^	5.84 (1.45–23.59)	0.013^*^	—		4.11 (1.69–10.01)	0.002^**^	7.28 (1.50–35.40)	0.014^*^
PTV location	0.81 (0.46–1.44)	0.47	0.76 (0.52–1.12)	0.17	0.51 (0.26–1.03)	0.06	—		—		—	
BED_10_							—		—			
< 90 Gy	1		1		1	0.46	—		—		1	
≥ 90 Gy	0.97 (0.19–4.86)	0.97	0.51 (0.20–1.33)	0.17	0.26 (0.07–0.98)	0.047^*^	—		—		0.11 (0.02–0.65)	0.015^*^
PTV compromise	0.54 (0.11–2.71)	0.46	1.37 (0.56–3.35)	0.49	2.02 (0.54–7.59)	0.30						
PTV	1.02 (1.00–1.05)	0.04^*^	1.02 (1.00–1.04)	0.012^*^	1.02 (1.01–1.05)	0.038^*^	0.99 (0.88–1.10)	0.80	1.01 (0.99–1.03)	0.40	0.94 (0.86–1.04)	0.25
ITV	1.06 (1.01–1.11)	0.03^*^	1.04 (1.01–1.08)	0.009^**^	1.05 (1.01–1.10)	0.03^*^	1.06 (1.01–1.11)	0.027^*^	1.00 (0.96–1.18)	0.86	1.01 (0.94–1.08)	0.86
RT regimen	—	0.95	—	0.72	—	0.70	—		—		—	

Abbreviations: BED_10_, biologically effective dose (withα/β ratio = 10); CI, confidence interval; ECOG, Eastern Cooperative Oncology Group; HR, hazard ratio; ITV, internal target volume; LC, local control; OS, overall survival; PFS, progression‐free survival; PTV, planning target volume; RT, radiation therapy.

^*^ *p* ≤ 0.05.

^**^ *p* ≤ 0.01.

^#^ Others refers to NSCLC, not otherwise specified (NOS).

### 2.5 Influence of Tumor Volume, T Stage, and Dose Intensity (BED10) on Outcomes

**FIGURE 3:**
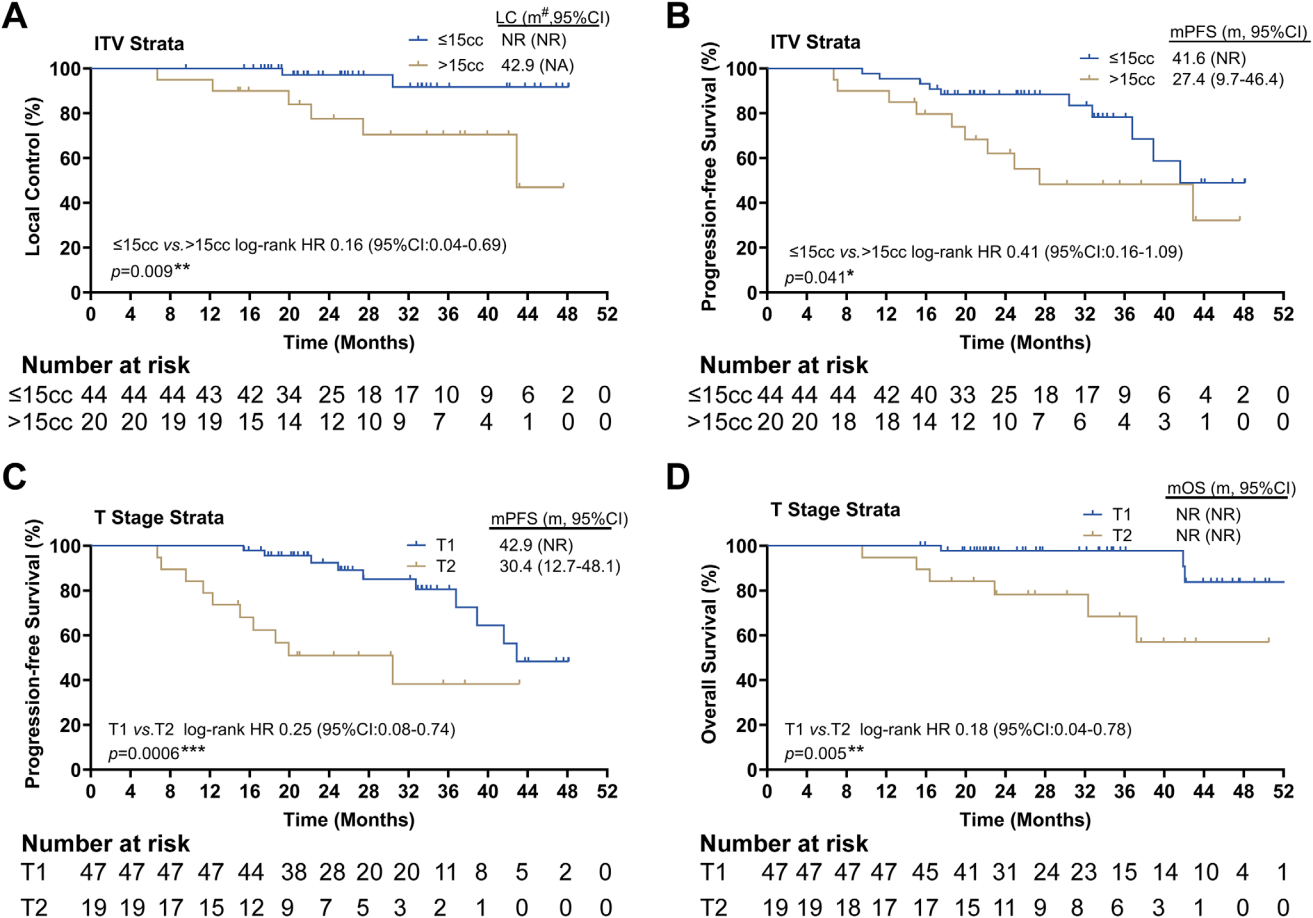
Stratified analysis of LC, PFS, and OS by ITV and T stage in the entire cohort. (A) Kaplan–Meier curves for LC by ITV (≤ 15 cc vs. > 15 cc) in the overall cohort. (B) Kaplan–Meier curves for PFS by ITV (≤ 15 cc vs. > 15 cc) in the overall cohort. (C) Kaplan–Meier curves for PFS by T stage (T1 vs. T2) in the overall cohort. (D) Kaplan–Meier curves for OS by T stage (T1 vs. T2) in the overall cohort. * *p* ≤ 0.05; ** *p* ≤ 0.01; ***p ≤ 0.001; ^#^m, months. CI, confidence interval; HR, hazard ratio; ITV, internal target volume; LC, local control; NA, not available; NR, not reached; OS, overall survival; PFS, progression‐free survival;.

Given the close correlation between T stage and tumor size, we performed further stratification analyses based on ITV size using receiver operating characteristic (ROC)‐derived thresholds (ITV ≤ 15 cc vs. ITV > 15 cc). Patients with ITV ≤ 15 cc exhibited significantly superior LC outcomes compared to those with ITV >15 cc (not reached [NR] vs. 42.9 months [m]; *p* = 0.009, Figure 3A). Similarly, a smaller ITV size (≤ 15 cc) was associated with significantly improved PFS (41.6 vs. 27.4 m; *p* = 0.041, Figure 3B). These findings highlight the consistently poorer outcomes observed with larger tumor volumes. Further stratification by T stage showed that T1‐stage patients experienced significantly longer median PFS (42.9 vs. 30.4 m; *p* = 0.0006) and superior OS (*p* = 0.005) compared to T2‐stage patients (Figure 3C,D), reinforcing the prognostic importance of tumor stage.

**FIGURE 4:**
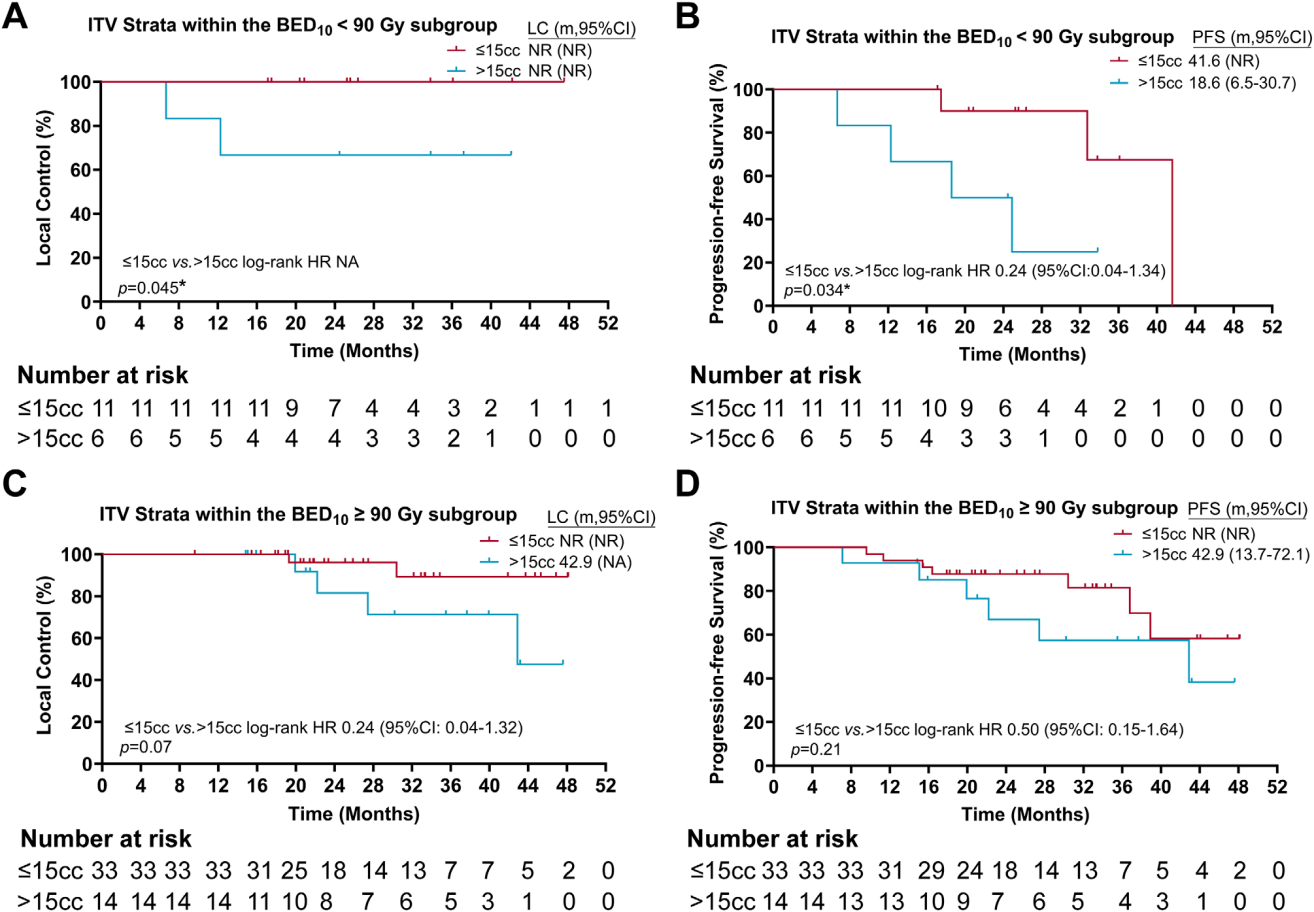
Stratified analysis of LC and PFS by ITV among patients with a BED_10_ < 90 Gy or ≥ 90 Gy subgroup. (A) Kaplan–Meier curves for LC by ITV (≤ 15 cc vs. > 15 cc) within the BED_10_ < 90 Gy subgroup. (B) Kaplan–Meier curves for PFS by ITV (≤ 15 cc vs. > 15 cc) within the BED_10_ < 90 Gy subgroup. (C) Kaplan–Meier curves for LC by ITV (≤ 15 cc vs. > 15 cc) within the BED_10_ ≥ 90 G subgroup. (D) Kaplan–Meier curves for PFS by ITV (≤ 15 cc vs. > 15 cc) within the BED_10_ ≥90 G subgroup. *p ≤ 0.05. BED_10_, biologically effective dose (with α/β ratio = 10); CI, confidence interval; HR, hazard ratio; ITV, internal target volume; LC, local control; NA, not available; NR, not reached; PFS, progression‐free survival.

We also investigated the influence of BED_10_ intensity (< 90 Gy vs. ≥ 90 Gy) on clinical outcomes. Across the entire cohort, no significant differences in LC or PFS were observed between the two BED_10_ groups (*p* = 0.16; Figure S5A,B). However, patients receiving BED_10_ ≥ 90 Gy demonstrated significantly better OS outcomes (Figure S5C). To further clarify the interaction between dose intensity and tumor size, we stratified patients within each BED_10_ subgroup by ITV size. In the BED_10_< 90 Gy subgroup, a smaller ITV size (≤ 15 cc) is significantly associated with improved LC (*p* = 0.045) and PFS (*p* = 0.034, Figure 4A,B), indicating that lower dose intensity may remain effective for smaller tumors. Conversely, within the BED_10_ ≥ 90 Gy subgroup, ITV size did not exert a significant effect on LC or PFS, though a non‐significant trend toward worse PFS was noted among larger tumors (Figure 4C,D). Importantly, ITV size had no impact on OS outcomes in either the BED_10_ subgroup (Figure S6).

Collectively, these findings highlight the prognostic significance of tumor volume, T stage, and BED_10_ intensity in UCNLC treated with SBRT. Our results underscore the potential benefits of early detection, accurate tumor size evaluation, and personalized SBRT approaches—including tailored dose adjustments and the strategic use of non‐uniform PTV compromises—to optimize therapeutic efficacy and safety.

## 3 Discussion

This study provides a comprehensive analysis of SBRT for UCNLC, presenting the largest single‐institution cohort to date (*n* = 66). Our findings indicate remarkable treatment outcomes, with 1‐ and 3‐year LC rates of 98.5% and 84.6%, respectively. PFS rates at 1‐ and 3‐year were 93.9% and 68.8%, respectively, while OS rates reached 98.5% at 1 year and 88.7% at 3 years. Notably, these OS outcomes exceed previously reported results from our institution in peripheral early‐stage NSCLC (88.3% at 1 year and 78.1% at 3 years), potentially due to the younger median age and greater tolerance of salvage therapies among these ultra‐central patients [17]. Treatment‐related toxicities were minimal, with only one patient (1.5%) experiencing grade 3 pneumonitis. Importantly, no patients experienced treatment‐related grade 4 or 5 adverse events, and no bronchopulmonary hemorrhages occurred, contrasting favorably with earlier UCNLC SBRT studies [12, 14, 15, 18, 19, 20, 21] (Table 4).

**TABLE 4:** Summary of outcomes and toxicity in selected SBRT studies using various radiotherapy regimens for ultra‐central tumors.

Study (year, *N*)	Definition of ultra‐central (UC)	Dose/fractionation	LC rate	OS rate	mPTV Dmax	≥ Grade 3 toxicities	Grade 5 toxicities	Reference
Chaudhuri et al. (2015, *n* = 34)^a^	GTV directly abutting the central airway	50 Gy/4‐5fx	100% 2 yr^b^	80% 2 yr	—	5.8%^c^	—	[18]
Tekatli et al. (2016, *n* = 47)	PTV overlapping the trachea or main bronchi	60 Gy/12fx		20.1% 3 yr	83.8 Gy/ 81.9 Gy	38%	15%	[19]
HILUS Study (2021, *n* = 65)	Tumors ≤ 1 cm from the main bronchi and trachea	56 Gy/8fx	83% 2 yr	—	97 Gy	33.8%	15.4%^d^	[11]
SUNSET Study (2024, *n* = 30)	PTV touches or overlaps the proximal bronchial tree, esophagus, pulmonary vein, or pulmonary artery	60 Gy/8fx	89.6% 3 yr	72.5% 3 yr	—	6.7%	3.3%	[14]
LungTech Study (2024, *n* = 31)	Tumors located ≤ 1 cm from the mainstem bronchi or the distal 2 cm of the trachea	60 Gy/8fx	81.5% 3 yr	61.1% 3 yr	—	6.5%, 19.4%^e^	3.2%	[13]
LUSTRE Study (2024, *n* = 30)^f^	Tumors that directly abut or overlap the PBT	60 Gy/8fx	85% 3 yr	58% 3 yr	116 Gy	17%	3.3%	[20]
Alexander et al. (2024, *n* = 56)	Tumors near critical structures (trachea, proximal tracheobronchial tree, esophagus, spinal cord, or heart) within 1 cm	60 Gy/15fx	—	—	—	3.7%^g^	1.9%^g^	[21]
Alexander et al. (2024, *n* = 56)	—	72 Gy/18fx	—	—	—	3.7%^g^	1.9%^g^	[21]

Abbreviations: GTV, gross tumor volume; LC, local control; OS, overall survival; PBT, proximal bronchial tree; PTV, planning target volume; SBRT, stereotactic body radiation therapy.

^a^ Mixed cohort (comparison between ultracentral and central tumors).

^b^ yr = year rates.

^c^ Mixed toxicity in the entire cohort; there are only seven patients with UCNLC.

^d^ Hemoptysis, *n* = 8, pneumonitis, *n* = 1; tracheoesophageal, fistula, *n* = 1.

^e^ Acute toxicity was 6.5%, and long‐term toxicity was 19.5%.

^f^ This is the secondary analysis of the LUSTRE phase III clinical trial.

^g^ The study encompassed central (*n* = 70) and ultra‐central (*n* = 56) tumors, with the incidence being assessed in the entire cohort. However, information on subgroup‐specific toxicity rates was not available.

These encouraging outcomes may be partly attributed to the stringent OAR constraints. Unlike previous trials, such as HILUS, where the omission of great vessel constraints may have contributed to fatal hemorrhages, our treatment planning emphasized rigorous volumetric dose constraints on critical structures, including the PBT, esophagus, heart, and great vessels, beyond traditional maximum point‐dose limitations. A recent secondary analysis of the LUSTRE trial supports our approach, demonstrating that patients experiencing grade 5 toxicity had notably higher volumetric dose exposures (D5cc, V90, and V100) to the PBT, despite comparable maximum point doses. Thus, volumetric dose parameters appear to influence late critical, high‐grade toxicities, reinforcing the importance of prioritizing volumetric dose parameters alongside minimizing Dmax in planning SBRT for ultra‐central lesions [20].

Another potential contributor to these favorable outcomes is the non‐uniform PTV strategy employed in cases with OAR abutted or overlapped. By implementing a PTVcrop or dividing the PTV into high‐ and low‐dose regions (PTV_high/ PTV_low), we effectively optimized dose delivery, maintained strict dose conformity, and preserved comprehensive tumor coverage. Specifically, the PTV_high volume received the full prescribed dose, while the PTV_low volume was ensured to receive at least 80% of this dose. To further enhance safety, the maximum dose hotspot was restricted to 120% of the prescribed dose, minimizing the risk of complications associated with SBRT in patients with UCNLC. This intentional dose heterogeneity enabled 53.3% of patients to safely receive 50 Gy in five fractions without experiencing severe toxicities, maintaining LC, and survival outcomes. The non‐uniform PTV approach thus represents a viable, safe, and effective solution for escalating doses while preserving treatment efficacy in UCNLC.

Our results also provide insight into prognostic factors for UCNLC treated with SBRT. Stratified analyses further revealed that a larger ITV (> 15 cc) was significantly correlated with inferior LC and PFS outcomes. Multivariate Cox regression analyses identified T stage as an independent predictor of PFS and OS, highlighting the prognostic importance of tumor size and stage. At the same time, radiation dose intensity (BED_10_) did not significantly affect LC and PFS across the entire cohort. Subgroup analyses revealed that smaller tumors (ITV ≤ 15 cc) achieved favorable LC and PFS outcomes even at BED_10_< 90 Gy. This finding suggests that small tumors may achieve relatively favorable outcomes under such dosing, while a reduced BED_10_ may not be optimal for large tumors. Conversely, patients with larger tumors benefited significantly from a non‐uniform PTV strategy, particularly regarding improved LC. These observations conceptually parallel the principles of spatially fractionated radiotherapy (SFRT), which utilizes heterogeneous dose distributions to enhance the therapeutic ratio by preserving normal tissue function while effectively controlling tumor growth [22, 23]. However, unlike traditional SFRT—which employs small high‐dose regions within larger tumors—our strategy expands high‐dose areas, ensuring comprehensive tumor coverage and improved conformity.

Despite the promising outcomes observed in our study, clinical results for larger tumors remain suboptimal. This underscores the need to integrate systemic strategies. Early evidence suggests that immunotherapy may augment the efficacy of SBRT in this setting. The Phase II trial (I‐SABR) reported a substantial 24% improvement in 4‐year event‐free survival (from 53% to 77%) when nivolumab was combined with SBRT compared to SBRT alone [24]. By contrast, a recent phase III study (NCT04214262) evaluating neoadjuvant, concurrent, and adjuvant atezolizumab with SBRT, presented at ASCO 2025, did not demonstrate a significant clinical benefit [25]; however, subgroup analyses specifically addressing larger tumors (> 4 cm) were not reported. Results from other ongoing Phase III trials (NCT03833154 and NCT03924869) are eagerly anticipated and are expected to clarify the further potential advantages of combining SBRT with immunotherapy [26, 27, 28]. Additionally, the ongoing I‐SABR‐SELECT Phase II trial, which employs deep learning to identify patients most likely to benefit from SBRT and immunotherapy combinations, has suggested particular advantages for larger tumors [29]. Given these insights, we propose that integrating immunotherapy with our non‐uniform PTV strategy may offer additional therapeutic advantages for larger ultra‐central tumors. Similar to SFRT, this approach might deliver heterogeneous radiation dose distributions, potentially increasing tumor immunogenicity, enhancing intratumoral perfusion, and alleviating hypoxia‑driven radioresistance. This combined strategy could foster a more favorable tumor microenvironment, thereby boosting tumor radiosensitivity and immunotherapy efficacy [30, 31]. In line with this hypothesis, our institution has initiated a prospective trial to evaluate the combination of SBRT and PD‐1 inhibitor serplulimab in early‐stage NSCLC, explicitly including those with larger ultra‐central lesions. Beyond underscoring the importance of meticulous radiation planning, respiratory motion management, and image guidance to deliver adequate SBRT doses for improved LC, future treatment paradigms should therefore prioritize personalized dose adjustments, integration of emerging technologies, and multidisciplinary strategies, including immunotherapy combinations and other systemic treatments, to optimize outcomes for larger ultra‐central tumors.

This study has several limitations that warrant consideration. First, it was a single‐center retrospective investigation, which introduces potential selection bias and limits the generalizability of our findings. Additionally, the relatively small sample size may have reduced the statistical power of subgroup analyses. Second, although the median follow‐up exceeded 32 months, longer observation is needed to fully capture late‐onset toxicities. Third, while the non‐uniform PTV strategy effectively reduced severe toxicities, it increases the workload complexity for medical physicists. Lastly, the generally small tumor sizes in our cohort likely contributed to the observed favorable outcomes; thus, validation in larger tumors is required to confirm efficacy more broadly. Addressing these limitations in future prospective, multi‑institutional studies will be critical to confirm our findings.

## 4 Conclusions

In conclusion, our findings highlight the importance of stringent OAR constraints in achieving optimal clinical outcomes for UCNLC patients. These results align with recently published guidelines from the American Radium Society, which emphasize the pivotal role of OAR constraints in UCNLC treatment [16]. We advocate for tailored non‐uniform PTV compromises to maximize OARs protection while maintaining effective local tumor control. By establishing safe dose thresholds for critical structures, we minimized the incidence of severe toxicities (grades 3–5) across various dose regimens. The division of PTV into high‐ and low‐dose regions appears to offer a balanced approach that enhances tumor control while mitigating damage to surrounding tissues. Although these findings are promising, further validation—incorporating larger sample sizes and advanced predictive models, including those powered by artificial intelligence —is essential. Such efforts will refine these strategies and potentially broaden their applicability in managing UCNLC with curative‐intent SBRT.

## 5 Materials and Methods

### 5.1 Study Cohort

This retrospective study, approved by the institutional ethics board of Shanghai Pulmonary Hospital, included 66 UCNLC patients who underwent SBRT between January 2020 and December 2023. All patients had histologically confirmed early‐stage (T1‐2N0M0) NSCLC, classified according to the eighth Edition American Joint Committee on Cancer Staging Manual [32]. Treatment‐naïve patients were either deemed medically inoperable by a thoracic surgeon or declined surgical intervention. Exclusion criteria included the presence of interstitial lung disease, regional lymph node metastasis, or tumor invasion into the bronchial lumen. All patients received 5–12 fractions, with total doses ranging from 40 to 70 Gy. Ultra‐central tumors were defined as those with PTV abutting or overlapping the PBT, esophagus, heart, great vessels, and pulmonary vessels, or internal target volume (ITV) within 1 cm of these structures (Figure 1A).

### 5.2 Non‐Uniform PTV Compromise and OAR Constraints

In cases where the PTV abutted central OARs, the PTV was tailored by creating a cropped target volume (PTVcrop), achieved by subtracting the OARs with an expansion margin of 1–3 mm. The prescription isodose surface was optimized to ensure that 95% of the PTVcrop was encompassed by the prescription isodose line, and 99% of the PTVcrop receives at least 90% of the prescribed dose (Figure 1B,E). When the PTV overlapped with central OARs, it was divided into PTV_high and PTV_low to prioritize OARs protection. For PTV_high, 95% of the volume was covered by the prescription isodose line, and 99% received at least 90% of the prescribed dose. For the PTV_low, 95% of the volume received at least 80% of the prescribed dose. The maximum dose within the PTV_high was constrained to approximately 120% at the normalization point (100%) to ensure that the dose within the ITV remained within the prescribed limits (Figure 1C, D and E). The maximum dose within the PTV_low was constrained to prevent exceeding the OARs' tolerance levels. In both methods, at least 95% of the PTV had to receive at least 80% of the prescribed dose.

In this study, we developed dose constraints for OARs based on their radiobiological characteristics, expressed as both physical doses (Gy) and equivalent doses in 2 Gy fractions (EQD2). Key OARs included the bronchus, trachea, esophagus, great vessels, spinal cord, heart/pericardium, and lung‐ITV. Dose metrics were defined by either maximum dose (Max*) or volumetric thresholds, and constraints were established for four distinct dose‐fractionation regimens: 50 Gy in 5 fractions, 60 Gy in 8 fractions, 60 Gy in 12 fractions, and 60 Gy in 15 fractions. All dose constraints for OARs across different fractionation regimens are documented in Table S1. These dose constraints were meticulously designed to optimize the therapeutic ratio in SBRT for UCNLC, effectively balancing tumor control with the protection of critical structures.

### 5.3 Radiation Therapy

All patients underwent four‐dimensional computed tomography (4D‐CT) scans to account for breathing motion. Respiratory gating was utilized for tumors with significant respiratory motion, particularly those located in the lower lobes of the lungs. Target volume and OARs were delineated, with a 5 mm margin applied to expand the ITV to PTV. Treatment planning was performed based on the average intensity projection of the 4D‐CT scans.

Treatment was delivered using intensity‐modulated radiotherapy (IMRT) with 6 MV flattening‐filter‐free (FFF) photon beams. To maintain precise target localization, position verification was performed prior to each fraction using online imaging modalities such as cone‐beam computed tomography (CBCT) or four‐dimensional CBCT (4D‐CBCT). Radiotherapy was administered on consecutive weekdays. Additional technical details of the SBRT procedure are provided in the Supporting Information Materials.

### 5.4 Study Outcomes and Data Collection

The primary endpoint of this study was LC and progression‐free survival (PFS). Secondary endpoints included regional control (RC), distant control (DC), OS, and SBRT‐related toxicities. Time‐to‐event outcomes were measured from the first day of SBRT. LC was determined by the absence of recurrence within the original PTV, whereas RC referred to the absence of recurrence in the same lung lobe or hilum/mediastinum, excluding the treated PTV. DC was characterized by the absence of metastatic disease in any extrathoracic location. PFS was defined as the interval from SBRT initiation until documented disease progression or death from any cause. OS was measured from the start of SBRT to death from any cause or the last known follow‐up, with censoring of patients lost to follow‐up. Treatment‐related toxicities were graded according to the National Cancer Institute—Common Terminology Criteria for Adverse Events version 5.0 [33], and tumor response was evaluated using the Response Evaluation Criteria in Solid Tumors version 1.1. All patients had comprehensive electronic records and imaging results documenting the treatment course, including baseline enhanced chest computed tomography (CT), brain magnetic resonance imaging (MRI), and positron emission tomography‐CT (PET‐CT). After treatment, chest CT scans, ultrasound of the clavicular region, and abdomen were conducted every 3 months during the initial 6 months, every 6 months in the subsequent 3 years, and annually thereafter. Brain MRI and bone scanning were performed annually. Acute toxicities were recorded within 6 months of SBRT, while long‐term toxicities were defined as those occurring more than 6 months after treatment.

### 5.5 Statistical Analysis

Descriptive statistics were used to summarize patient characteristics. Continuous variables were reported as medians with 95% confidence intervals (CIs), while categorical variables were presented as counts and percentages. The Kaplan–Meier method was employed to estimate LC, PFS, and OS. Differences in survival between groups were assessed using the log‐rank test. Potential prognostic factors for LC, PFS, and OS were first screened in univariable Cox models. All variables with *p* < 0.05 in univariable analyses were then included in a multivariable Cox regression model with backward stepwise selection, retaining only those factors with *p* < 0.05. Hazard ratios (HRs) and their 95% CIs were calculated to quantify the strength of association. All statistical analyses were performed using SPSS version 24 (IBM Corp., Armonk, NY, USA). A two‐sided *p*‐value < 0.05 was considered statistically significant. Significance levels are indicated as follows: *p* < 0.05; *p* < 0.01; *p* < 0.001.

## Author Contributions

Di Liu: project administration, data curation, writing – original draft, writing – review and editing. Shuangyan Yang: project administration, data curation, writing – original draft, writing – review and editing. Yun Chen: data curation, investigation, writing – review and editing. Ming Liu: data curation, investigation, writing – review and editing. Ruifeng Zhao: data curation, investigation, writing – review and editing. Minren Hu: data curation, investigation, writing –review and editing. Bin Su: data curation, investigation, writing – review and editing. Qiongya Wu: data curation, investigation, writing – review and editing. Hongyu Wu: data curation, investigation, writing – review and editing. Hui Liu: methodology. Jinming Yu: conceptualization, supervision, data curation, writing – review and editing. Yaping Xu: conceptualization, supervision, data curation, writing – review and editing. All authors have read and approved the final manuscript.

## Funding

The study is supported by the Science and Technology Commission of Shanghai Municipality (21DZ2201900 and 24Y12800301), Shanghai Pulmonary Hospital (Clinical research key project: FKLY20006), and Beijing Bethune Charitable Foundation (YDTR‐007 and YDTR‐008).

## Ethics Statement

This study was approved by the institutional research ethics board of Shanghai Pulmonary Hospital (approval number: K21‐312Y). Informed consent was obtained from all participants in the study.

## Conflicts of Interest

The authors declare no conflicts of interest.

## Supporting information




**Supplementary Table S1**: Dose constraints for OARs across different fractionation regimens
**Supplementary Table S2**: Characteristics of Patients Who Experienced Local Failure After SBRT
**Supplementary Table S3**: The efficacy of SBRT in UCNLC
**Supplementary Figure S1**: Heatmap of Acute and Long‐Term Toxicities in the Study Cohort
**Supplementary Figure S2**: Comparative Dose‐Volume Histogram (DVH) Analysis Between the Non‐Uniform PTV Subgroup and the Normal PTV Subgroup
**Supplementary Figure S3**: Subgroup Analysis of LC, PFS, and OS Stratified by PTV compromise
**Supplementary Figure S4**: Subgroup Analysis of PFS and OS Stratified by BED10 in the Normal PTV Group Without Compromise
**Supplementary Figure S5**: Subgroup Analysis of LC, PFS, and OS Stratified by BED10 in the Entire Cohort
**Supplementary Figure S6**: Stratified analysis of OS by ITV among patients with a BED10 <90 Gy or ≥ 90 Gy subgroup.

## Data Availability

All data used and/or analyzed in the study are available in the supplementary materials or from the corresponding author upon reasonable request.
