# Supplementary material for: Precision Stereotactic Body Radiotherapy for Ultra‐Central NSCLC: Tailored Margins Reduce Toxicity Without Compromising Tumor Control
Source: MedComm (2020). 2026 Jul 22;7(8):e70589. doi: 10.1002/mco2.70589 (PMC13390614; doi:10.1002/mco2.70589)
Supplement: Supplementary file 1 — Supplementary Table S1: Dose constraints for OARs across different fractionation regimens Supplementary Table S2: Characteristics of Patients Who Experienced Local Failure After SBRT Supplementary Table S3: The efficacy of SBRT in UCNLC Supplementary Figure S1: Heatmap of Acute and Long‐Term Toxicities in the Study Cohort Supplementary Figure S2: Comparative Dose‐Volume Histogram (DVH) Analysis Between the Non‐Uniform PTV Subgroup and the Normal PTV Subgroup Supplementary Figure S3: Subgroup Analysis of LC, PFS, and OS Stratified by PTV compromise Supplementary Figure S4: Subgroup Analysis of PFS and OS Stratified by BED10 in the Normal PTV Group Without Compromise Supplementary Figure S5: Subgroup Analysis of LC, PFS, and OS Stratified by BED10 in the Entire Cohort Supplementary Figure S6: Stratified analysis of OS by ITV among patients with a BED10 <90 Gy or ≥ 90 Gy subgroup. [file MCO2-7-e70589-s001.pdf]

Precision Stereotactic Body Radiotherapy for Ultra-Central NSCLC: Tailored Margins Reduce Toxicity Without Compromising Tumor Control

Di Liu[1#], Shuangyan Yang[1#], Yun Chen[1], Ming Liu[1], Ruifeng Zhao[1], Minren Hu[1], Bin Su[1], QiongYa Wu[1], Hongyu Wu[1], Hui Liu[1], Jinming Yu[2*], Yaping Xu[1*]

[#]: Contributed equally; [*]: Joint correspondence authors

[1] Department of Radiation Oncology, Shanghai Pulmonary Hospital, School of Medicine, Tongji University, Shanghai, China.

[2] Department of Radiation Oncology, Shandong Cancer Hospital and Institute, Shandong First Medical University and Shandong Academy of Medical Sciences, Jinan, China.

Correspondence

Yaping Xu, Department of Radiation Oncology, Shanghai Pulmonary Hospital, School of Medicine, Tongji University, Shanghai, China; No.507 Zhengmin Road, Yangpu District, Shanghai; E-mail: 1800515@tongji.edu.cn;

Jinming Yu, Department of Radiation Oncology, Shandong Cancer Hospital and Institute, Shandong First Medical University and Shandong Academy of Medical Sciences, Jinan, PR China. No.440 Jiyan Road, Jinan City, Shandong Province; E-mail: sdyujinming@163.com.

Di Liu and Shuangyan Yang contributed equally to this paper.

1. Supplementary Methods

2. Supplementary Table 1-3

3. Supplementary Figures 1-6 and Legends

1. Supplementary Methods

1.1 Patient Simulation

Positioning

Patients undergoing SBRT are positioned in the supine position with both arms elevated above the head. To ensure the reproducibility of the position during treatment and to minimize intrafractional motion, various immobilization devices may be used, including vacuum bags and/or body molds.

For cases involving significant respiratory motion (exceeding 0.5 cm), it is critical to employ motion management techniques to ensure accurate target localization. Evaluation of tumor motion should be conducted during the 4D CT simulation. In our institution, respiratory gating was used most for motion management.

Image Acquisition

A 4D-CT scan is required to evaluate and account for respiratory-induced tumor motion. 4D-CT scan is conducted after stabilizing the patient's respiratory pattern, with the respiratory motion monitored using a real-time position management (RPM) system. Intravenous (IV) contrast is mandatory. The 4D-CT dataset includes images from multiple respiratory phases, typically binned into 10 phases, covering the complete respiratory cycle (0%-90%). The CT images have a slice thickness of 2.5 mm to ensure high-resolution data for treatment planning. The scan range should extend from 1 cm above the lower border of the mandible to the lower border of the second lumbar vertebra.

Target Volume Delineation

For tumors located entirely within the lung parenchyma, recommended window/level settings are: 800–1600 HU (window width), and -600 to -750 HU (window level). For lesions adjacent to the mediastinum, the recommended mediastinal window settings used to evaluate and adjust the target volume are: 350 – 400 HU (window width), and 20 – 40 HU (window level), considering potential invasion of the mediastinum and surrounding organs. The gross tumor volume (GTV) should encompass the primary tumor along with any spiculation and pleural involvement. If the maximum intensity projection (MIP) clearly defines the tumor boundaries, the internal target volume (ITV) is delineated on the MIP and checked across all respiratory phases to ensure full tumor coverage. When the MIP lacks sufficient clarity, GTVs are manually delineated on each phase and accumulated into a composite ITV. And PTV is created by adding an anisotropic margin of 5 mm to the ITV to account for potential uncertainties related to setup variation and residual motion.

Organs at Risk (OARs) Delineation

The following OARs for planning should be contoured on the average intensity projection (AVG) image set:

Spinal Cord: The spinal cord is contoured based on its bony boundaries within the spinal canal, starting from the inferior border of the cricoid cartilage (for apical lung tumors, contouring should begin at the base of the skull) down to the inferior border of lumbar spinal (L2), contouring slice by slice, excluding the neural foramina.

Esophagus: Contour on mediastinal windows, from the starting point at the inferior border of the cricoid cartilage down to the esophagogastric junction.

Brachial Plexus: For upper lobe tumors, only the ipsilateral brachial plexus needs to be contoured on mediastinal or head & neck windows. This includes the spinal nerves from C4/5 (C5 nerve root) to T1/2 (T1 nerve root) passing through the neural foramina.

Heart: Contour along the pericardium, starting from the pulmonary artery at the midline plane at the base (or upper part) and extending down to the apex of the heart, on mediastinal windows.
Great Vessels (Includes the aorta, pulmonary artery, and vena cava): The great vessels should be contoured using mediastinal windows, including the vessel wall, muscle layer, and perivascular fat (with an additional 5 mm margin for contrast-enhanced vessel walls). The great vessels should be contoured slice by slice, extending 3 cm above and below the PTV.

Trachea and Proximal Bronchial Tree: Include the distal 2 cm of the trachea, carina, bilateral main bronchi, bilateral upper lobe bronchi, intermediate bronchus, right middle lobe bronchus, lingular bronchus, and bilateral lower lobe bronchi. Contour the lobar bronchi up to the bifurcation of the segmental bronchi. They should be defined on lung windows.

Ribs/Chest Wall: The chest wall can be contoured by expanding 2 cm outward from the ipsilateral lung in the lateral, posterior, and anterior directions. The contour extends anteriorly to the edge of the sternum and posteriorly to the vertebral bodies, including the area where the spinal nerve roots emerge. The 2 cm chest wall contour includes the intercostal muscles and nerves but excludes the vertebrae, sternum, and skin. The rib contour can also be limited to 3 cm beyond the PTV for the relevant tissue.

Lungs: Both lungs should be contoured using the pulmonary windows, including all areas of inflammation, fibrosis, and atelectasis. Small vessels extending beyond the hilum (with a diameter less than 1 cm) should also be included. The ITV (specifically the intrapulmonary ITV), hilum, and trachea/main bronchi should be excluded from the whole lung structure.

1.2 Treatment Planning

Beam Energy and treatment techniques

TrueBeam (Varian Medical System, Palo Alto, CA, USA) with 6MV FFF mode is preferred as it allows for higher dose rates, significantly reducing treatment time. In our study, we employed static IMRT, with beam arrangement based on the tumor location. Typically, 9–11 coplanar beams were used, with ipsilateral beam arrangement to minimize the contralateral lung radiation dose.

Dose optimization

The prescription dose is determined based on the clinical target volume and treatment objectives. Due to the heterogeneity of patient tissues, dose calculations should employ Type-B algorithms or Monte Carlo (MC) simulations. The Eclipse treatment planning system uses the Acuros XB advanced dose calculation algorithm, and a dose grid resolution of 0.125 cm × 0.125 cm × 0.125 cm is recommended for optimal accuracy in dose distribution.

Prescription Dose and Coverage

The prescription dose in this study was tailored based on the tumor's location and its proximity to critical OARs. Several dose-fractionation schemes were employed, including 50 Gy in 5 fractions, 60 Gy in 8 fractions, 60 Gy in 12 fractions, and 60 Gy in 15 fractions. For all treatment plans, the goal was to ensure that at least 95% of the planning target volume (PTV) was covered by 100% of the prescribed dose ($V100\% \geq 95\%$), and at least 99% of the PTV received 90% of the prescribed dose ($V90\% \geq 99\%$). However, when the PTV abutted central OARs, a cropped PTV (PTVcrop) was created by subtracting the OAR with 1 mm expansion, and in cases when the PTV overlapped with

central OARs, the PTV was subdivided into two regions: PTV_high and PTV_low. PTV_high was defined as the PTV minus OAR with a 1 mm expansion, and the remaining part was defined as PTV_low (Figure 1). In such cases, intentional compromises in PTV with a lower prescription dose in PTV_low were made to ensure that OARs remained within their dose tolerance limits.

For PTV_high, the dose was carefully controlled, with a maximum dose constraint of 120% at the normalization point (100%), meaning the highest dose was limited to 120% of the prescribed dose. This strategy ensured that the dose within the ITV, which accounts for tumor motion, remained within safe limits while delivering an optimal therapeutic dose to the tumor. For PTV_low, the priority was to ensure that 80% of the prescribed dose was covered at least 95% of the volume, balancing tumor control with protection of OARs.

Conformity and Low Dose Spillage

For ultra-central lung tumors, the conventional metrics for dose conformality and dose fall-off may not be fully applicable due to the use of non-uniform target volumes in SBRT. In these cases, the commonly used indices, such as conformality and dose spillage metrics, require adjustments to better reflect the specific challenges associated with treating tumors located in close proximity to critical structures.

The conformality of the high-dose volume can still be evaluated using the ratio of the prescription isodose (100%/80% of the prescription dose) volume to the PTV_high/PTV_low volume (R100%). Intermediate and low-dose spillage are assessed with the R50%, which represents the ratio of the 50% prescription isodose volume (100%/80% prescription dose) to the PTV_high/PTV_low volume. Additionally, the dose delivered at a 2 cm distance from the PTV (D2cm) can also be used to monitor dose fall-off. However, due to the unique anatomical and dosimetric challenges of ultra-central lung tumors, stricter or modified criteria may be necessary to balance effective tumor control with the need to minimize dose exposure to nearby OARs.

Dose Limits for OARs

The dose constraints for OARs were evaluated using the equivalent dose in 2 Gy fractions (EQD2) (Table S1), allowing for comparisons across different fractionation regimens while maintaining isotoxicity. These constraints ensured that the dose received by the OARs was within safe thresholds, minimizing the risk of severe complications such as grade 3-5 toxicities.

Plan QA and criteria

For SBRT patients, Plan QA was performed prior to therapy to ensure the accelerator's performance meets criteria, requiring a γ passing rate of at least 95% with 3%/2mm.

1.3 Position Verification

Position verification will be performed before the first day of patient treatment. Prior to each treatment session, kV CBCT or 4D CBCT (in the case of gating treatment) should be used for image-guided verification. Initially, registration should be performed based on bony anatomy, followed by soft tissue alignment to the ITV. For tumors located near OARs, OAR contours and the corresponding dose avoidance lines should be transferred from the planning system to the CBCT console. The recommended tolerances for translational shifts (x, y, z) should be within $\leq$3 mm, and rotational errors (pitch, roll, yaw) should be limited to $\leq$1 degree. For translational errors greater than 3 mm but less than 5 mm, or rotational errors exceeding 1 degree, a position correction should be made and verified by CBCT

re-imaging. If the translational error exceeds 5 mm, or the rotational error exceeds 3 degrees, a full re-setup and re-imaging are necessary to confirm proper positioning. If more than two instances of translational errors greater than 5 mm or rotational errors exceeding 3 degrees occur during the entire treatment course, a re-setup and replanning are strongly recommended.

2. Supplementtray Tables

Supplementary Table S1 Dose constraints for OARs across different fractionation regimens

OARs	α/β	Metric	EQD2(Gy)	Constraints (Gy)			
				50Gy/5fx	60Gy/8fx	60Gy/12fx	60Gy/15fx
Bronchus	3	Max*	63.36	33.00	39.75	46.22	50.00
		< 0.5 cc	30.24	21.00	24.79	28.24	30.17
Trache	3	Max*	88.00	40.00	48.53	56.86	61.80
		< 4 cc	20.79	16.50	19.23	21.63	22.95
Esophagus	10	Max*	49.58	35.00	39.75	43.63	45.61
		< 5 cc	22.59	19.50	21.39	22.78	38.00
GreatVessels	3	Max*	144.16	53.00	64.88	76.73	83.88
		< 10 cc	116.56	47.00	57.33	67.54	73.66
SpinalCord	2	Max*	43.75	25.00	30.26	35.36	38.37
		< 0.35 cc	37.95	23.00	27.75	32.33	35.01
		< 1.2 cc	17.76	14.50	17.14	19.56	20.92
Heart/pericardium	3	Max*	80.56	38.00	46.02	53.81	58.41
		< 15 cc	60.16	32.00	38.50	44.72	48.86
Lung-ITV	3	>1000cc	15.39	13.50	15.56	17.32	18.25
		>1500cc	13.75	12.50	14.35	15.91	16.72
		MLD	5.04	6.00	6.59	7.03	7.24
		< 10%	28.00	20.00	23.55	26.76	28.55

* Defined as 0.035cc or less; Refer to AAPM TG 101 (50 Gy/5 Fx). Abbreviations: OAR, Organ at Risk; EQD2, Equivalent Dose in 2 Gy fractions; fx, fractionation; Max， Maximum Dose; ITV, Internal Target Volume; MLD, Mean Lung Dose

Supplementary Table S2 Characteristics of Patients Who Experienced Local Failure After SBRT

**Patient**	**Age**	**Sex**	**Histology**	**Tumor size**	**OARs**	**LC time (m*)**	**PTV compromise (Y/ N)** [#]	**BED_{10} (Gy)**
No.1	79	Female	Adeno*	2.1cm	PBT	42.9	No	100
No.2	64	Male	Adeno	2.8cm	PBT	27.4	No	100
No.3	73	Female	NSCLC	3.7cm	PBT	30.4	Yes	100
No.4	73	Male	Adeno	3.3cm	PBT	12.3	No	86.4
No.5	77	Female	Adeno	2.8cm	PBT	22.2	Yes	100
No.6	81	Male	NSCLC	3.6cm	PBT	19.9	No	100
No.7	64	Male	Adeno	3.1cm	PArtery*	6.7	No	76.8
No.8	75	Male	Adeno	1.6cm	Heart	19.2	Yes	100

*Adeno=Adenocarcinoma; m=months; PArtery=Pulmonary Artery. [#] Y/N=Yes/ No
Abbreviations: OAR, Organ at Risk; LC, Local Control; m, months; PTV, Planning Target Volume; BED10, Biologically Effective Dose (withα/β ratio = 10); PBT, Proximal Bronchial Tree.

Supplementary Table S3 The efficacy of SBRT in UCNLC

**Efficacy**	**Median (95%CI, m*)**
LC	NR*
PFS	42.9 (36.1-49.7)
OS	NR*
**Tumor Response**	**N (%)**
CR	6 (9.1)
PR	46 (69.7)
SD	14 (21.2)
PD	0
DCR	66 (100)

*m=months; NR=Not Reached.

Abbreviations: SBRT, Stereotactic Body Radiation Therapy; UCNLC, UCNLC, Ultra-central Non-small Cell Lung Cancer; CI, Confidence Interval; LC, Local Control; PFS, Progression-free Survival; OS, Overall Survival; CR, Complete Response; PR, Partial Response; SD, Stable Disease; PD, Progressive Disease; DCR, Disease Control Rate.

3. Supplementary Figure Legends

Supplementary Figure S1 Heatmap of Acute and Long-Term Toxicities in the Study Cohort

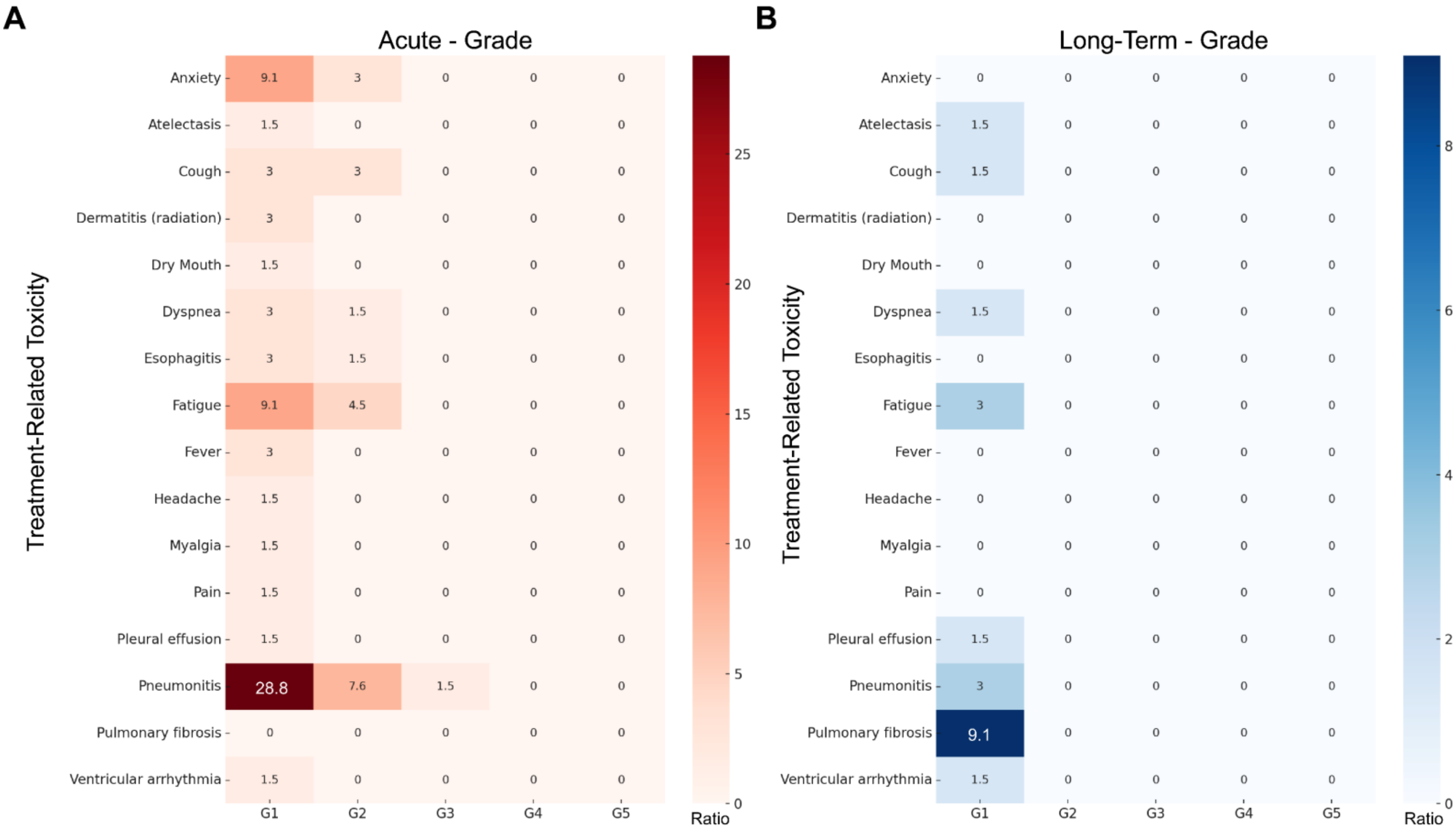

Figure S1

A: Incidence of acute toxicity in UCNLC patients treated with SBRT.
B: Incidence of long-term toxicity in UCNLC patients treated with SBRT.

Supplementary Figure S2 Comparative Dose-Volume Histogram (DVH) Analysis Between the Non-Uniform PTV Subgroup and the Normal PTV Subgroup

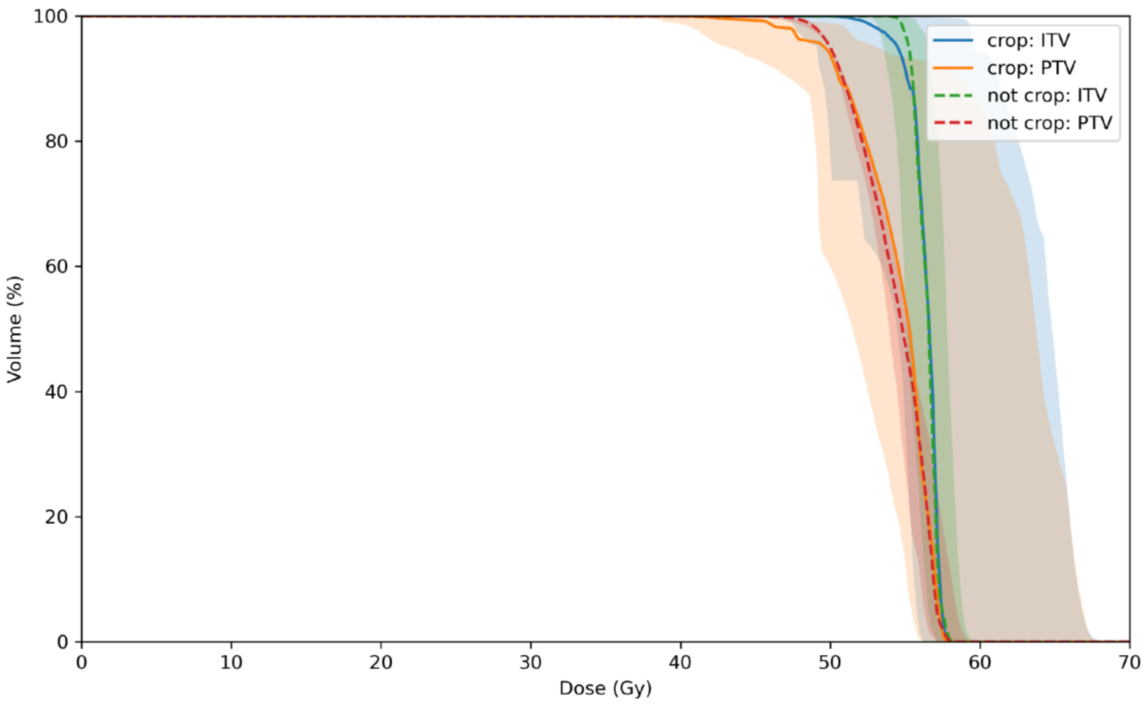

Figure S2

The term "crop" refers to the non-uniform PTV strategy, which includes the settings of PTVcrop and PTV_high/PTV_low; conversely, "non-crop" denotes the normal PTV subgroup.

Abbreviations: ITV, Internal Target Volume; PTV, Planning Target Volume.

Supplementary Figure S3 Subgroup Analysis of LC, PFS, and OS Stratified by PTV compromise

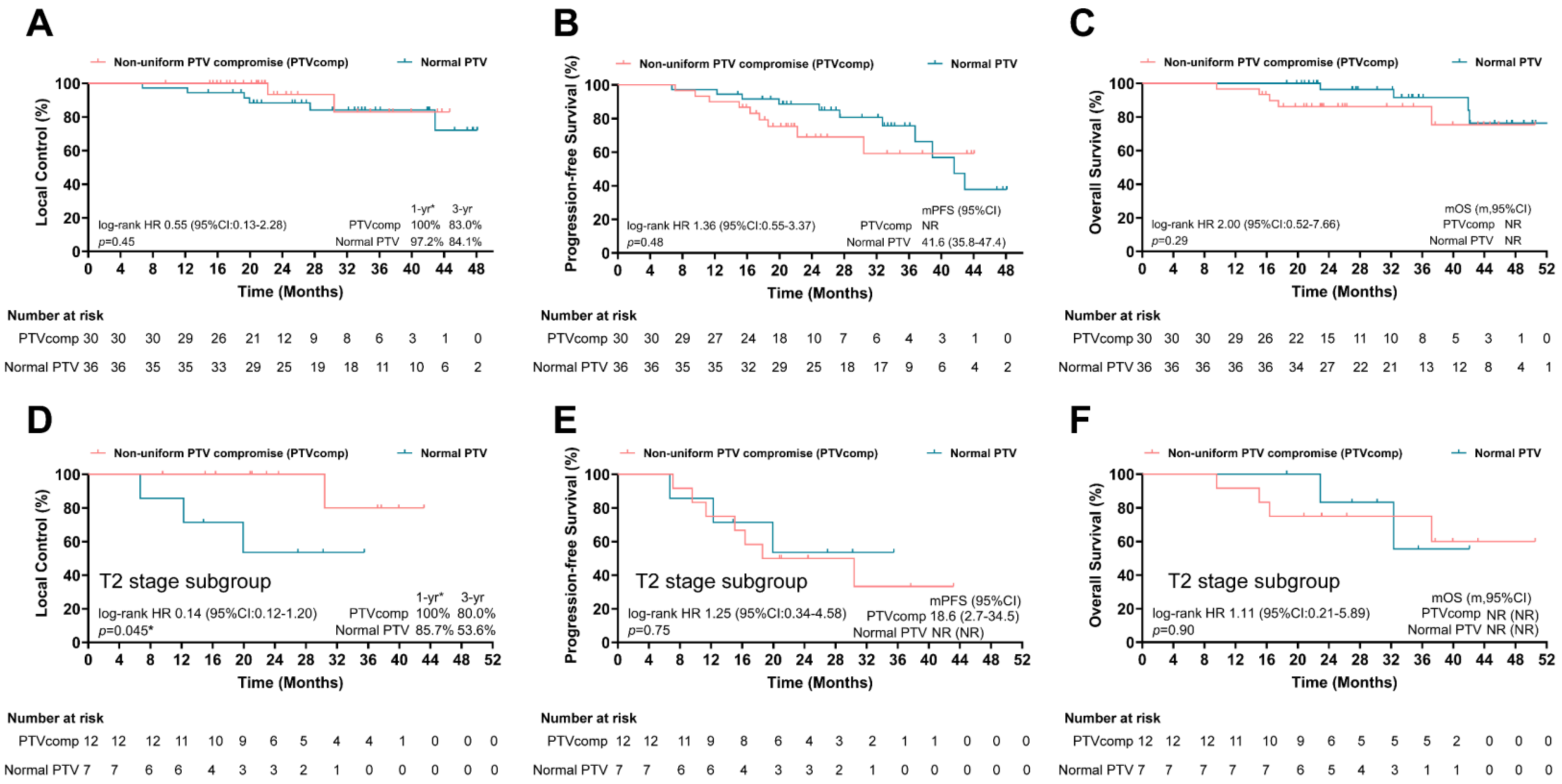

Figure S3

Kaplan-Meier plots showing LC (A), PFS (B), and OS (C) stratified by PTV compromise. No significant differences were observed between the non-uniform PTV compromise group and the normal PTV group.

Kaplan-Meier plots showing LC (D), PFS (E), and OS (F) stratified by PTV compromise in the T2 stage subgroup.

*yr=year rates. **p* $\leq$ 0.05; Abbreviations: PTV, Planning Target Volume; HR, Hazard Ratio; CI, Confidence Interval; PFS, Progression-free Survival; NR, Not Reached; OS, Overall Survival.

Supplementary Figure S4 Subgroup Analysis of PFS and OS Stratified by BED_{10} in the Normal PTV Group Without Compromise

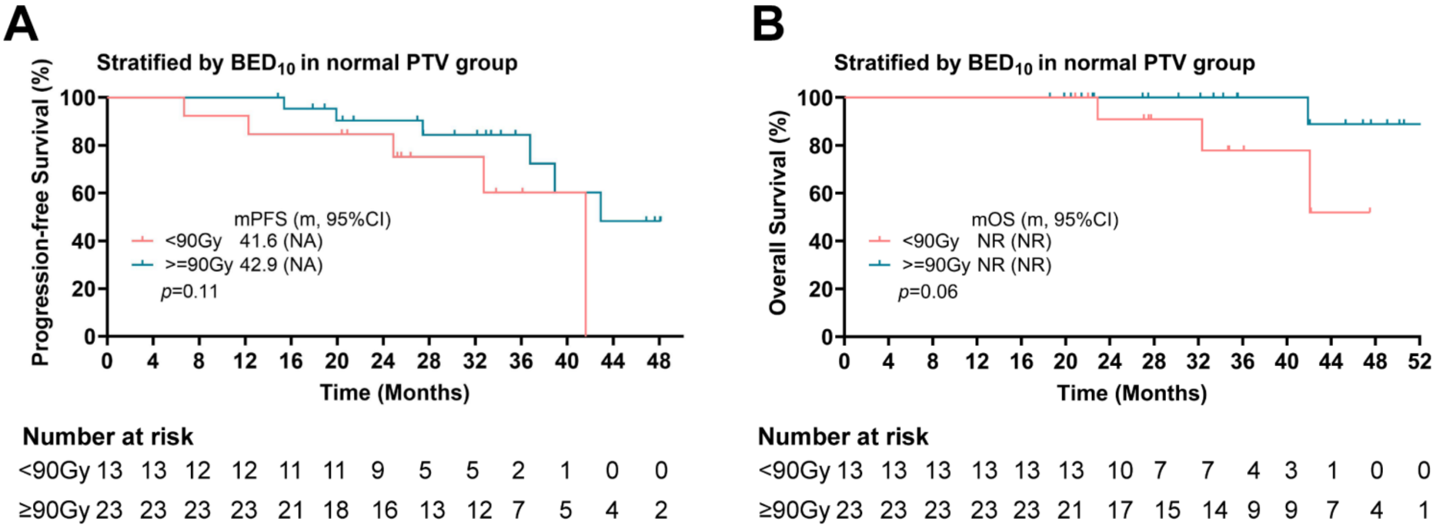

Figure S4

A: Kaplan-Meier plots showing PFS (A) and OS (B) stratified by BED_{10} (< 90 Gy vs. ≥ 90 Gy) in the normal PTV group without compromise.

Abbreviations: BED_{10}, Biologically Effective Dose (withα/β ratio = 10); PTV, Planning Target Volume; CI, Confidence Interval; NA, Not Available; NR, Not Reached; HR, Hazard Ratio; PFS, Progression-free Survival; OS, Overall Survival.

Supplementary Figure S5 Subgroup Analysis of LC, PFS, and OS Stratified by BED_{10} in the Entire Cohort

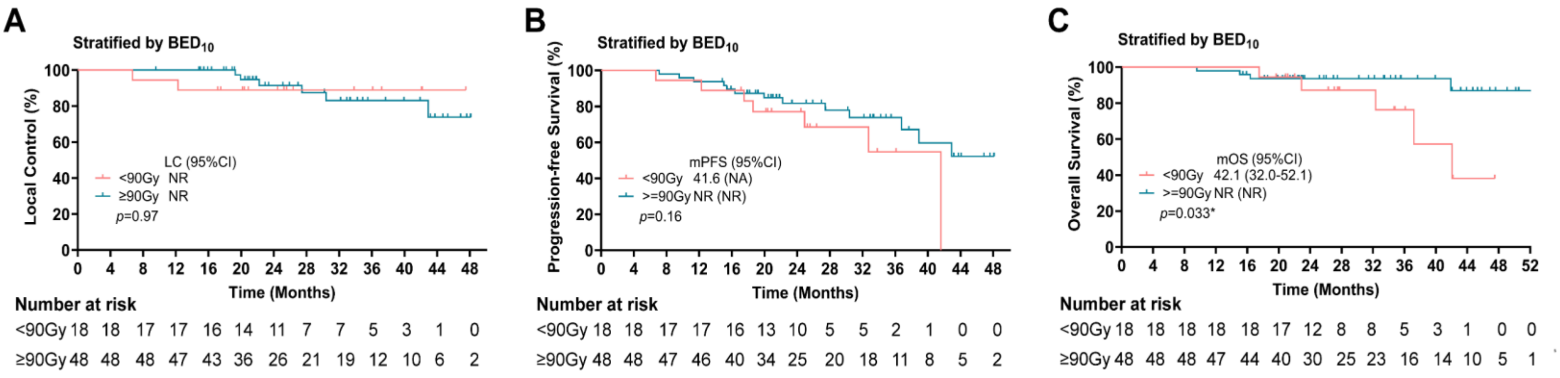

Figure S5

Kaplan-Meier plots showing LC (A), PFS (B), and OS (C) stratified by BED_{10} (<90 Gy vs. ≥90 Gy) in the entire cohort.

Abbreviations: BED_{10}, Biologically Effective Dose (with α/β ratio = 10); LC, Local Control; CI, Confidence Interval; NR, Not Reached; PFS, Progression-free Survival; NA, Not Available; OS, Overall Survival.

Supplementary Figure S6 Stratified analysis of OS by ITV among patients with a BED_{10} <90 Gy or ≥ 90 Gy subgroup

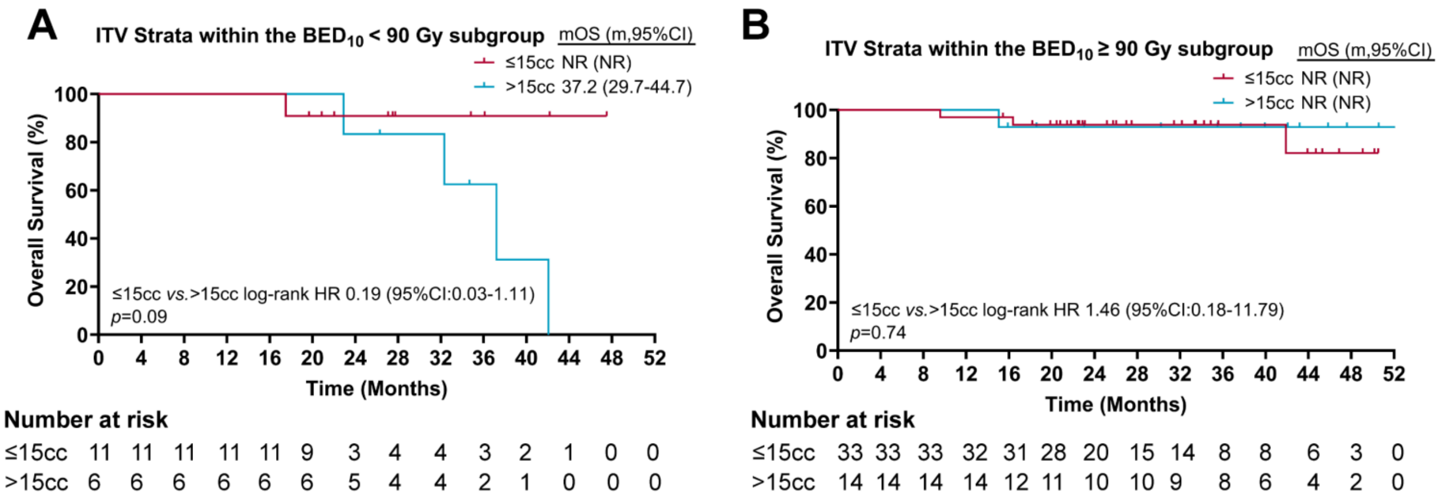

Figure S6

A: Kaplan–Meier curves for OS by ITV (≤ 15 cc vs. > 15 cc) within the BED_{10} < 90 Gy subgroup.
B: Kaplan–Meier curves for OS by ITV (≤ 15 cc vs. > 15 cc) within the BED10 ≥ 90 Gy subgroup.

Abbreviations: ITV, Internal Target Volume; BED_{10}, Biologically Effective Dose (with α/β ratio = 10); OS, Overall Survival; CI, Confidence Interval; NR, Not Reached; HR, Hazard Ratio.
